# Fabrication of polyamide-12/cement nanocomposite and its testing for different dyes removal from aqueous solution: characterization, adsorption, and regeneration studies

**DOI:** 10.1038/s41598-022-16977-8

**Published:** 2022-07-30

**Authors:** Saleh Ahmed Aldahash, Prerna Higgins, Shaziya Siddiqui, Mohammad Kashif Uddin

**Affiliations:** 1 Department of Mechanical and Industrial Engineering, College of Engineering, Majmaah University, Al-Majmaah, 11952 Kingdom of Saudi Arabia; 2 Department of Chemistry, Sam Higginbottom University of Agriculture Technology and Sciences, Prayagraj, U.P. 211007 India; 3 Department of Chemistry, College of Science, Al-Zulfi Campus, Majmaah University, Al-Majmaah, 11952 Kingdom of Saudi Arabia

**Keywords:** Environmental sciences, Chemistry, Engineering, Materials science, Nanoscience and technology

## Abstract

Polyamide-12/Portland cement nanocomposite was prepared by using the exfoliated adsorption method. The fabricated nanocomposite was applied first time to remove Congo red (CR), brilliant green (BG), methylene blue (MB), and methyl red (MR) from the synthetic wastewater. The polymer nanocomposite was characterized by Fourier transform infrared spectroscopy, scanning electron microscopy, energy-dispersive X-ray spectroscopy, elemental mapping, Brunauer–Emmett–Teller surface area analysis, and X-ray diffraction. The adsorption was rapid and all the studied dyes were absorbed on the surface of the polymer nanocomposite in 90 min. The point of zero charge was found at pH 5 and the factors such as pH, time, and temperature were found to affect the adsorption efficiency. Freundlich isotherm and pseudo-second-order models well-fitted the adsorption isotherm and kinetics data, respectively. The calculated maximum adsorption capacity was 161.63, 148.54, 200.40, and 146.41 mg/g for CR, BG, MB, and MR, respectively. The mode of the adsorption process was endothermic, spontaneous, and physical involving electrostatic attraction. On an industrial scale, the high percentage of desorption and slow decrease in the percentage of adsorption after every five regeneration cycles confirm the potential, practicality, and durability of the nanocomposite as a promising and advanced adsorbent for decolorization of colored wastewater.

## Introduction

The advancement in water treatment technologies by preparing new and efficient nanomaterials is useful to overcome the shortcomings of traditional adsorbents. This will lead to the discovery of materials with increased and better adsorptive performance. Therefore, research on the development of novel nanocomposites for use in water purification has been popular and in constant demand^1–8^. The synthesis of nanocomposites is accomplished through various methods, and they are successfully used to remove aqueous pollutants such as Ni/ZnO/g-C_3_N_4_^9^, Co@ZnO^10^, Cu–ZnO/S-g-C_3_N_4_^11^, TiO_2_/Graphene oxide^12^, AI-MnO^13^ and propionic acid treated bagasse^14^.

Polyamide-12 (PA-12) with the chemical formula (C_12_H_23_NO)_n_ is a semi-crystalline thermoplastic polymer with superior mechanical and thermal properties. A successful material because of its ideal characteristics and many applications, PA-12 has excellent chemical resistance and tensile strength. PA-12 has numerous applications in plastic manufacturing processes, and in the metal coatings, sport, automotive and electrical industries. In the last decades, polyamide (PA) has attracted scientists in the development of new materials with an interesting perspective and huge potential in many industrial fields such as wastewater treatment. PA-12 microspheres have shown remarkable adsorption properties for triclosan, an antibacterial agent, in water^15^. PA-12 facilitated rapid adsorption and removed 98% of triclosan. Bassyouni et al. fabricated nanofibrous Fe_3_O_4_/o-MWCNTs/polyamide-6 membrane for the removal of Pb (II) ions^16^. As prepared electrospun novel hybrid nanocomposite exhibited easy regeneration and separation properties. In another study, electrospun fibers with polyamide-6 and chitosan shell were synthesized and used for the removal of an antibiotic, i.e., tetracycline^17^. For the desalination purpose, a polyamide nanofiltration membrane was prepared through interfacial polymerization and electrostatic assembly by assembling a nontoxic organic compound, i.e., phytic acid on it and their removal efficiency was checked^18^. Recent reviews have highlighted the properties of polyamide desalination membranes in large-scale desalination and membrane separation of toxic organic compounds^19,20^. Saleh et al. have used titania-incorporated polyamide nanocomposite^21^, silica/polyamide nanocomposite^22^, polyamide embedded magnetic palygorskite^23^, polyamide-graphene composite^24^, polyamide grafted carbon microspheres^25^, and clay-based polyamide nanocomposites^26^ for the adsorption of dyes and toxic metals.

Textile industries use dye products for coloration. The discharged dyes from the cloth industries in water streams are huge toxin chemicals that contain poisonous ingredients such as corrosive alkali and reducing agents^27^. Many dyes don’t degrade in water. Some are carcinogenic, and are banned in several European countries. Congo red (CR) is an anionic, organic azo dye. It appears as a red-brown color which turns red in the alkali solution and blue in the acidic solution. CR produces harmful effects even at very low concentrations and thus is highly toxic. Brilliant green (BG) dye is a cationic, organic dye of the malachite-green dye series. BG is used as an antiseptic drug and antibacterial agent in the diluted solution. BG has toxic and mutagenic effects on the gastrointestinal tract which may lead to organ damage with long exposure^28^. Methylene blue (MB) is a thiazine cationic dye. MB has antimicrobial applications. In the form of medicine, MB is used to treat melanoma and methemoglobin levels^29^. MB is known to cause severe central nervous system problems. At high doses (< 2 mg/kg), MB can cause cardiac and pulmonary vascular issues, and renal and mesenteric blood flow^30^. At doses (> 5 mg/kg), MB may precipitate fatal serotonin in the body^31^. Methyl red (MR) is a benzoic azo dye. It is not easily decomposed in aqueous streams and leads to serious environmental problems. It was found that the toxicity of MR was almost three to fivefold higher near pH 6^32^.

For many decades, adsorption process has been the most famous and most used wastewater treatment technique for removing aqueous pollutants^33–35^. Recently, the removal of CR^36,37^, BG^38,39^, MB^40,41^, and MR^42,43^ from colored aqueous solution was conducted successfully. PA-12 is new in the area of environmental science. Portland cement (PC) is a hydraulic material that consists primarily of different compounds of calcium silicates (2CaO·SiO_2_, 3CaO·SiO_2_, 3CaO·Al_2_O_3_, 4CaO·Al_2_O_3_Fe_2_O_3_) and clay minerals (SiO_2_, Al_2_O_3,_ and Fe_2_O_3_). It has been used as a low-cost adsorbent^44–47^. PA incorporated with PC has improved mechanical properties and cost benefits. Gadelmoula and Aldahash evaluated the tensile, compressive, and flexural strength of selective laser sintering (SLS) manufactured PA-12/white cement parts^48^. It was found that a 10% concentration of PC in the mixture was sufficient to improve mechanical properties of PA-12 sintered specimens^49^. Another study showed that the addition of PA enhanced PC’s mechanical properties by significantly reducing mechanical cement fatigue by 93.3%^50^. Yuan et al. found that the addition of hot-melt polyamide to hydraulic cement led to excellent crack healing properties and improvement in flexural strength^51^. A further study also showed that adding PA fiber increased the tensile and flexural strength of aggregate concrete^52^.

In this study, a polymer nanocomposite of PA-12 and PC was prepared and tested for the adsorption of four different dyes’, i.e., CR, MG, MB, and MR. According to the literature survey we conducted, the as-prepared nanocomposite was utilized for the first time for the adsorption of targeted dyes. As PC is cheaper than PA-12, the prepared nanocomposite is less expensive than pure PA-12. PC has a porous surface area and is hence used in this study to reduce the cost of synthesized nanocomposite by decreasing the quantity of PA-12. The aim was to synthesize a nanocomposite that has properties such as low-cost and easy preparation that required low mechanical resources. The as-prepared nanocomposite (PA-12/PC nanocomposite) has many advantages such as high surface area, remarkable adsorption properties, ease to use, and cost-effectiveness. The influence of some important factors affecting the adsorption of the dyes was monitored and the isotherm and kinetic modeling and thermodynamic studies of the adsorption process were studied under optimum conditions. The adsorption results were found to be excellent for CR, BG, MB, and MR. Considering its desorption and regeneration utility, the prepared novel PA-12/PC nanocomposite is promising for industrial wastewater treatment.

## Results and discussion

### Characterization

**Figure 1:**
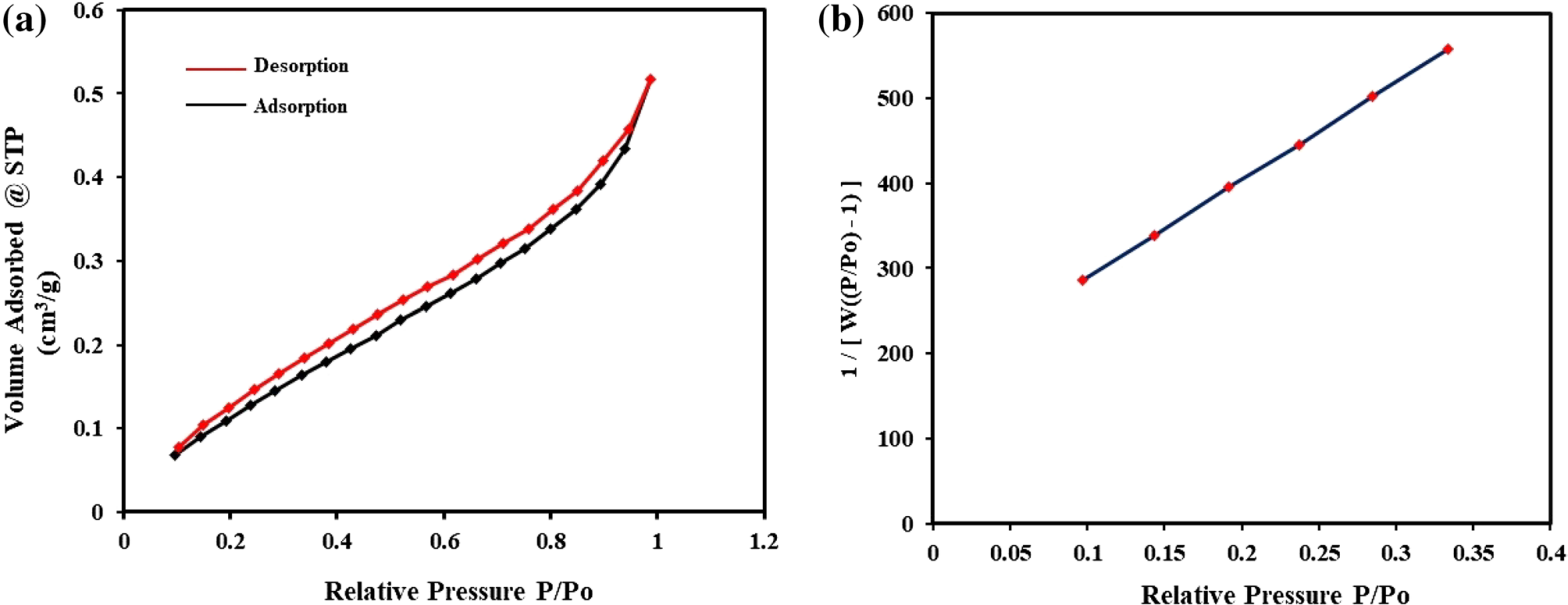
BET adsorption–desorption isotherm (**a**) A plot of 1/[W(P/P_0_ − 1)] vs. (P/P_0_) (**b**).

The Brunauer–Emmett–Teller (BET) method has been used in nanoscience for several decades now. This method is considered best in the determination of the surface area of the material. A H4 hysteresis loop of type-II isotherm with nearly horizontal and parallel lines over a wide *p*/*p*° range has been observed (Fig. 1a), which indicates that the pore shape of PA-12/PC nanocomposite is of microporous characteristics. Type H4 loops are usually shown by many nanoporous adsorbents^53^. Type-II isotherm belongs to successive multilayer formation processes during the adsorption process^54^. A linear BET multipoint plot of 1/[W(P/P_0_) − 1] vs P/P_0_ (Fig. 1b) was obtained (R^2^ = 0.999) and their slope was used to calculate the specific surface area. The BET surface area, total pore volume, and average pore diameter were 2.635 m^2^/g, 0.003 cm^3^/g, and 2.665 nm, respectively. The values suggest the structure characteristics of PA-12/PC nanocomposite as a microporous solid with a relatively small external surface. The small BET surface area and the pore volume of PA-12/PC nanocomposite may be due to the occlusion of internal micropores by incorporating PA-12 in PC. Gedam and Dongre also reported the decrease in porosity and specific surface area of the prepared composite due to the blockage of internal pores by iodate upon doping with chitosan^55^. The mean pore diameter of PA-12/PC nanocomposite was small, leading to a high adsorption performance towards CR, BG, MB, and MR molecules. Recently, Liu et al. also analyzed that the smaller pore diameter of polymeric adsorbent (primary aminated resin) caused higher equilibrium adsorption capacity for phenol and anionic surfactant^56^.

**Figure 2:**
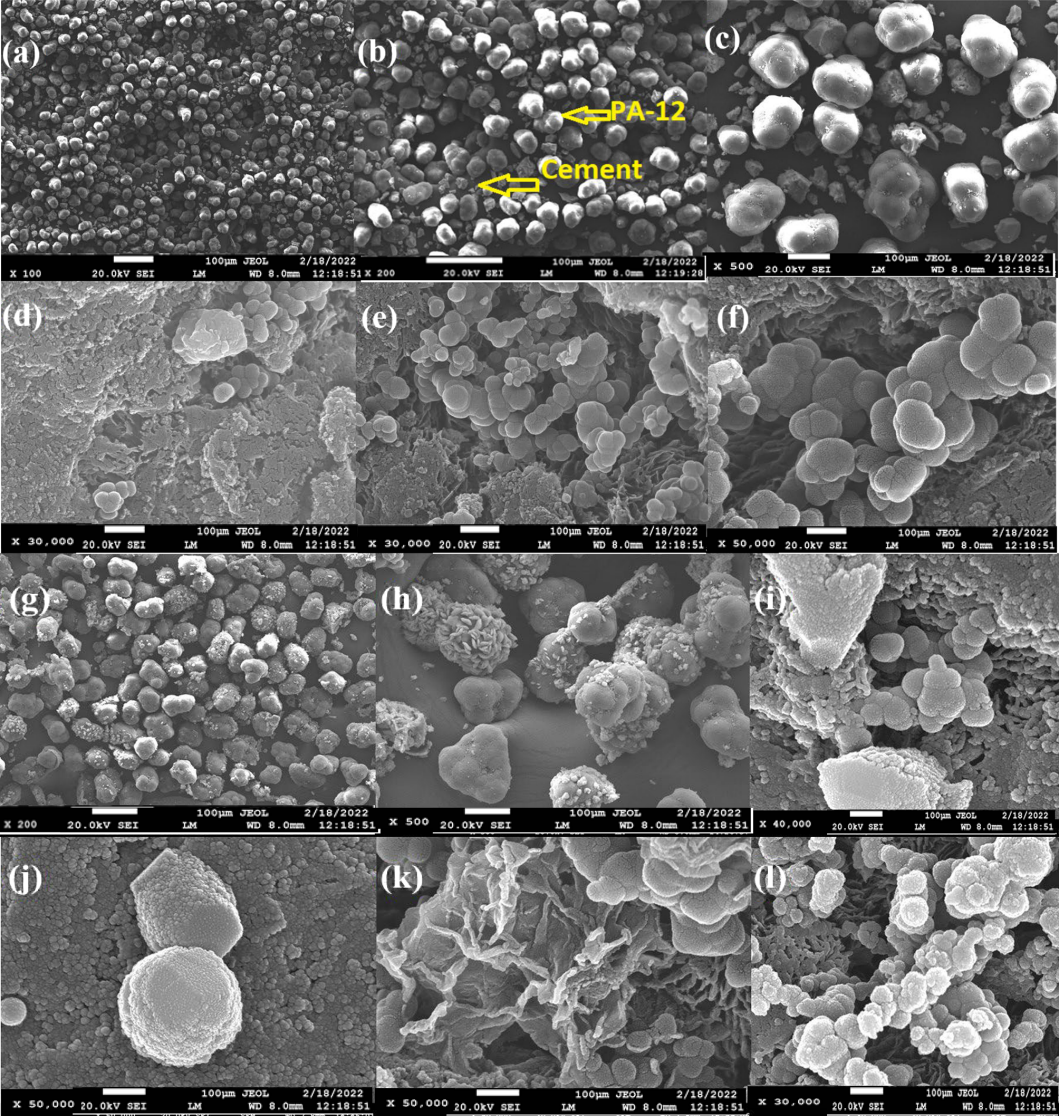
SEM images of PA-12/PC nanocomposite (**a–f**), CR adsorbed PA-12/PC nanocomposite (**g,h**), BG adsorbed PA-12/PC nanocomposite (**i,j**), MB adsorbed PA-12/PC nanocomposite (**k**) and MR adsorbed PA-12/PC nanocomposite (**l**).

Figure 2a–k shows scanning electron microscopy (SEM) images of PA-12/PC nanocomposite before and after dyes’ uptake. Figure 2a–c show that PA-12 particles are round in shape while PC particles are angular. It can also be noted that PA-12 clusters are surrounded by PC particles. Evenly distributed clusters of PA-12 in the form of clumps confirm its uniform distribution all over the composite. The PA-12 surface is spherical, soft, fibrous, and granular while the PC particles combine to bundle together to form larger particles which are homogeneously dispersed in the structural network of PA-12/PC nanocomposite (Fig. 2d–f). It can be seen that PC particles cover the round PA-12 particles and some of the PA-12 particles are visible while most of the PA-12 particles are encapsulated in PC confirming the formation of a nanocomposite (Fig. 2d–f). After dyes’ adsorption, the surface of PA-12/PC became brighter. CR was seen arranged onto the adsorbents’ surface in the grain shape (Fig. 2g,h). BG was adsorbed in the cluster form (Fig. 2i,j), and MB adsorption created new links between strands of PA-12/PC nanocomposite (Fig. 2k) and Fig. 2l proves the MR deposition onto PA-12/PC nanocomposite.

**Figure 3:**
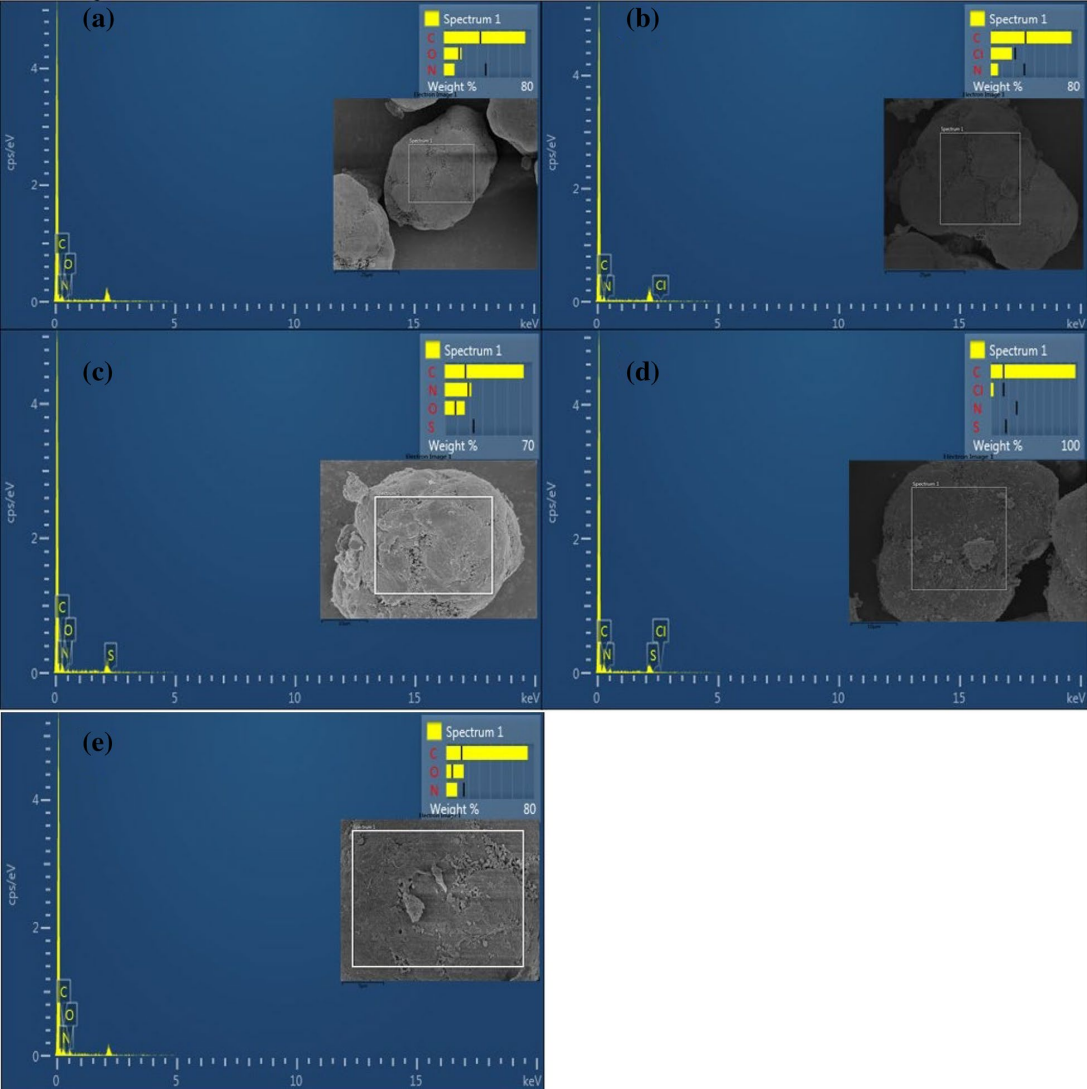
EDS images of PA-12/PC nanocomposite (**a**), CR adsorbed PA-12/PC nanocomposite (**b**), BG adsorbed PA-12/PC nanocomposite (**c**), MB adsorbed PA-12/PC nanocomposite (**d**) and MR adsorbed PA-12/PC nanocomposite (**e**).

**Figure 4:**
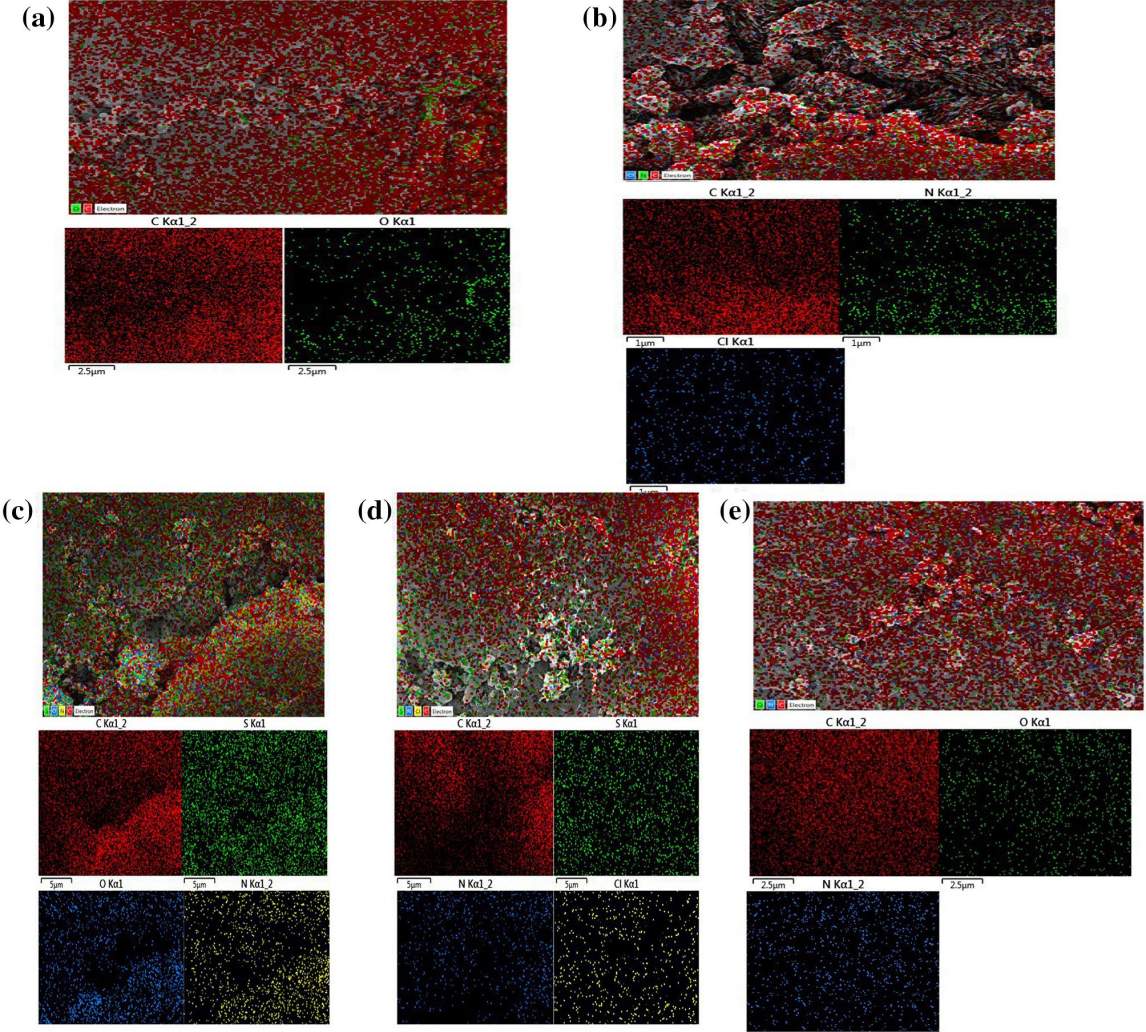
Elemental mapping of PA-12/PC nanocomposite (**a**), CR adsorbed PA-12/PC nanocomposite (**b**), BG adsorbed PA-12/PC nanocomposite (**c**), MB adsorbed PA-12/PC nanocomposite (**d**) and MR adsorbed PA-12/PC nanocomposite (**e**).

The element analysis of the PA-12/PC nanocomposite by energy dispersive spectroscopy (EDS) is shown in Fig. 3a–e. As carbon (C) element is the main constituent of both PA-12 and PC, it was detected with a sharp peak in the PA-12/PC nanocomposite (Fig. 3a). It can also be seen in Fig. 3a, that PA-12/PC nanocomposite is composed of carbon (74.33 weight percent), oxygen (16.14 weight percent), and nitrogen (9.53 weight percent) which are uniformly distributed on the surface of PA-12/PC nanocomposite. In CR adsorbed PA-12/PC nanocomposite, chlorine (22.41 weight percent) is determined (Fig. 3b), sulfur (23.05 weight percent) in BG adsorbed PA-12/PC nanocomposite (Fig. 3c), chlorine (14.71 weight percent) and sulfur (17.29 weight percent) are determined in MB adsorbed PA-12/PC nanocomposite (Fig. 3d), carbon (78.45 weight percent), nitrogen (8.99 weight percent) and oxygen (12.56 weight percent) are determined in MR adsorbed PA-12/PC nanocomposite (Fig. 3e) along with their respective weight percentage changes confirm the dyes’ successful adsorption as evidenced by the additional peaks in EDS figures. The surface distribution of the detected elements on PA-12/PC nanocomposite can be seen in the element maps (Fig. 4a–e).

**Figure 5:**
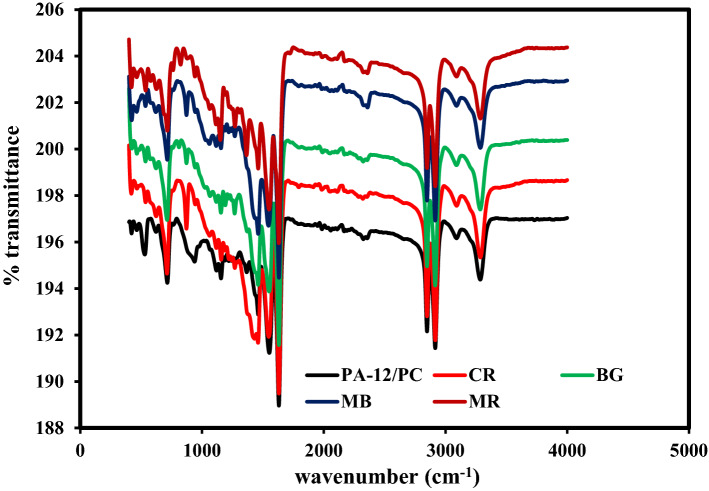
FT-IR spectra of PA-12/PC before and after dyes adsorption.

Fourier transform infrared spectroscopy (FTIR) spectra of PA-12/PC nanocomposite (Fig. 5) show a broad peak at 3285.56 cm^−1^, which is attributed to hydrogen-bonded N–H stretching^57^. The peaks recorded at 2915.66 cm^−1^ and 2848.30 cm^−1^ belong to symmetrical and asymmetrical stretching of C–H^21^. Peak observed at 1631.50 was related to C=O stretching vibration of the amide I^58^ while narrow peaks at 1553.01 and 1461.58 cm^−1^ were ascribed to N–H bending and C–N stretching vibrations of the amide II^59^, respectively. The sharp peak at 1156.30 cm^−1^ carrying a small peak at 938.90 cm^−1^ might be correlated to Si–O–C stretching. The peak at 715.21 cm^−1^ could be attributed to the substituted aromatic rings. The sharp peak at 530.36 cm^−1^ commonly corresponds to O–Si–O bending^60^ while the peak at 421.44 cm^−1^ belongs to the Zn–O–Si group^61^. The FTIR spectra of raw PA-12 (Fig. S1a) and PC (Fig. S1b) were also examined to confirm the functional groups existing in the prepared PA-12/PC nanocomposite and it can be seen that many characteristic absorption peaks for PA-12 and PC particles were observed in the produced PA-12/PC nanocomposite. As a result of dyes’ adsorption, new peaks at 2359, 1366, 1270, 1191, 824, and 872 cm^−1^ were detected which indicates that dyes’ molecules are involved on the surface of PA-12/PC nanocomposite. Additionally, most of the adsorption peaks remained unchanged, suggesting that the interaction of dyes’ molecules with PA-12/PC nanocomposite did not alter the functional groups of the adsorbent after adsorption, since they were physically adsorbed by the PA-12/PC nanocomposite.

**Figure 6:**
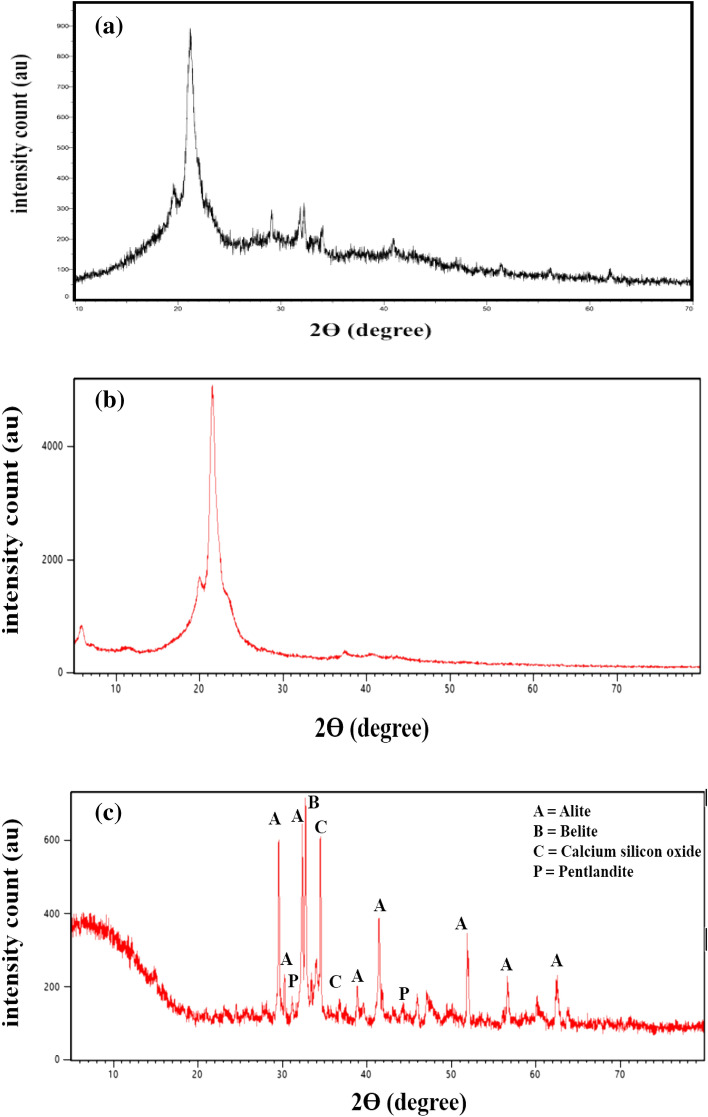
XRD pattern of PA-12/PC nanocomposite (**a**), PA-12 powder (**b**) and PC (**c**).

The X-ray diffraction pattern (XRD) of PA-12/PC nanocomposite is present in Fig. 6a. According to general observation, the composite seems to possess a slight degree of crystallinity, exhibiting many sharp PC and PA-12 peaks. For PA-12, both α and γ phases were observed in the PA-12/PC nanocomposite. The slight peak at ~ 11° and a distinct peak at 21.2° ~ 2θ correspond to the γ-phase, while the peak at 19.8° ~ 2θ corresponds to the α-phase. The pronounced peak with a peak area from 20 to 25° ~ 2θ suggests the formation of the α-phase during precipitation^62,63^. Apart from these, the peaks of calcium silicates are indicative of the presence of PC at higher ~ 2θ as observed by Ribeiro et al.^64^ and Jaya et al.^65^. The crystalline structures of PA-12 powder (Fig. 6b) and PC (Fig. 6c) were also analyzed  by using XRD pattern. PA-12 powder is a semi crystalline polymer exhibiting two distinct peaks at 20.9° ~ 2θ and 22.0° ~ 2θ which correspond to the unstable and intermediate structures of α and γ phase^66^ (Fig. 6b). The XRD pattern of the PC provides some insight into its elemental composition. Most of the intensity peaks are of impure forms of common silicates, i.e., alite (Ca_3_SiO_5_) and belite (Ca_2_SiO_4_) while other peaks of aluminate, pentlandite and oxides are also found^67,68^ (Fig. 6c).

**Figure 7:**
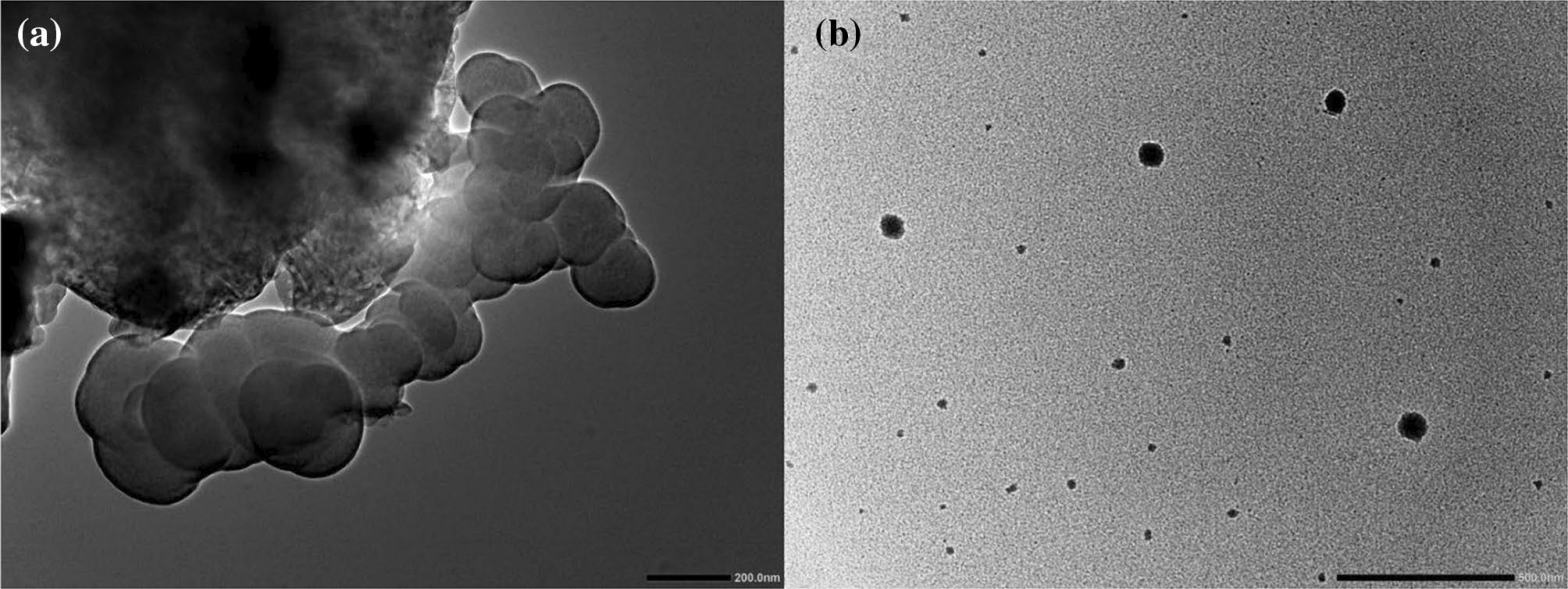
(**a,b**) HRTEM of PA-12/PC nanocomposite.

From the high resolution transmission electron microscopy (HRTEM) images, it can be concluded that PA-12 particle are spherical and wrapped on the surface of PA-12/PC nanocomposite in the form of globular agglomeration (Fig. 7a). The distribution of the PA-12 particles in the matrix of PA-12/PC nanocomposite is reasonably good and the inter-particle distance of PA-12 is fairly uniform (Fig. 7b).

### Adsorption studies

#### Effect of pH and adsorption mechanism

**Figure 8:**
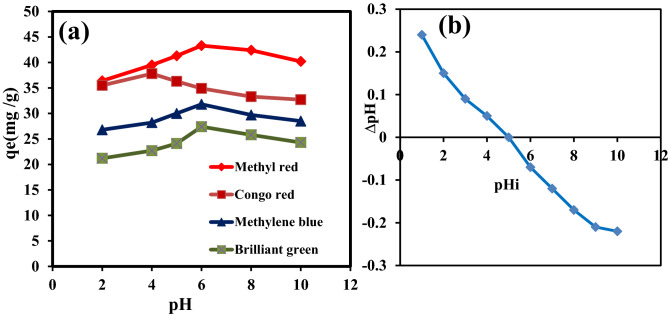
Effect of pH on CR, BG, MB and MR adsorption onto PA-12/PC nanocomposite (**a**) point of zero charge of PA-12/PC nanocomposite (**b**).

**Figure 9:**
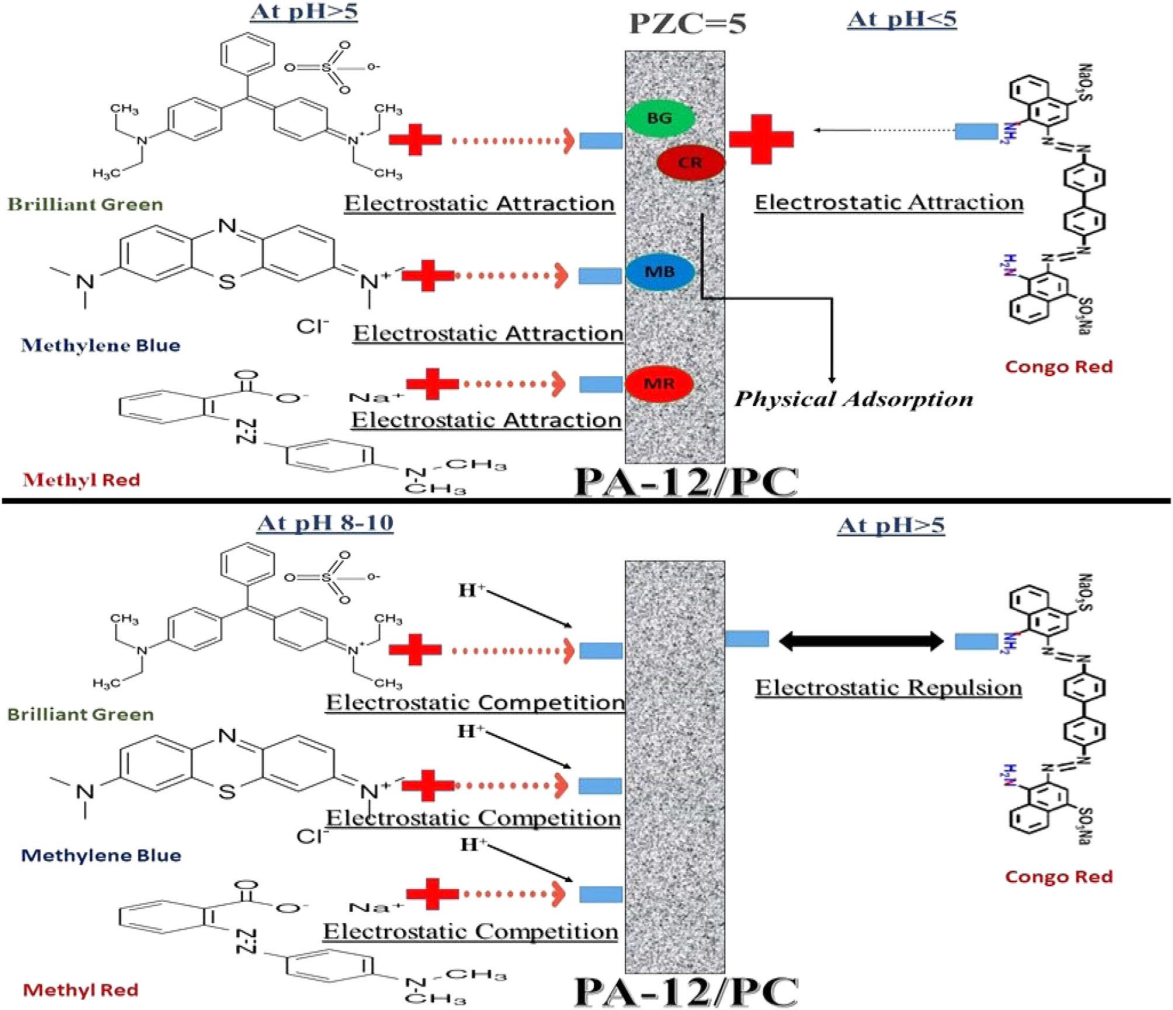
Adsorption mechanism of dyes adsorption on PA-12/PC nanocomposite.

pH is a significant factor to be accounted for in the adsorption study as it influences the surface binding sites and adsorption capacity of the adsorbent. Figure 8a shows the effect of different pH values on the adsorption of CR, BG, MB, and MR onto PA-12/PC nanocomposite. The results can be explained by the point of zero charge (PZC) value. PZC is a point where the value of ΔpH (pH_*f*_ − pH_*i*_) becomes zero and below this point, the adsorbent has a positively charged surface, whereas, above this point, the adsorbent has a negatively charged surface. The PZC value of PA-12/PC nanocomposite came out at pH 5 (Fig. 8b), and therefore below and above this pH, PA-12/PC nanocomposite has positive and negative charged surfaces, respectively. With regard to CR, equilibrium adsorption capacity (qe) increases slightly with pH increasing from 2 to 4, reaches a maximum at pH 4, and then decreases with pH increasing from 5 to 10 (Fig. 8a). CR, an anionic dye, holds a negative sulfonate (SO_3_^−^) group in its chemical structure. Therefore, electrostatic attraction between the positively charged surface of PA-12/PC nanocomposite (pH < 5) and negatively charged CR results in adsorption, while from pH 5 to 10, there is electrostatic repulsion between anionic charges of both PA-12/PC nanocomposite and CR, resulting in decreased adsorption. Recent studies have also reported pH 4 as an optimum pH for CR adsorption^69,70^. For other cationic dyes, i.e., BG, MB, and MR, qe increases with increasing pH from 2 to 6 and then decreases slightly with increasing pH from 8 to 10. The reason for low qe at pH < PZC is the competitiveness of dissociated H^+^ ions with BG, MB, and MR adsorption at positive surface sites of PA-12/PC nanocomposite. With increasing pH, negative adsorption sites become available and adsorption reached the maximum at pH 6. At pH 8–10, adsorption again decreases because of the presence of OH^−^ ions in the solution which leads to precipitation of dye molecules and disrupts the bonding between PA-12/PC nanocomposite and BG, MB, and MR. Several studies have also reported that pH 6 is the optimum pH for the adsorption of BG^71^, MB^72^, and MR^73^. Figure 9 shows the adsorption mechanism of CR, BG, MB, and MR adsorption on PA-12/PC nanocomposite.

#### Effect of contact time and initial concentration

**Figure 10:**
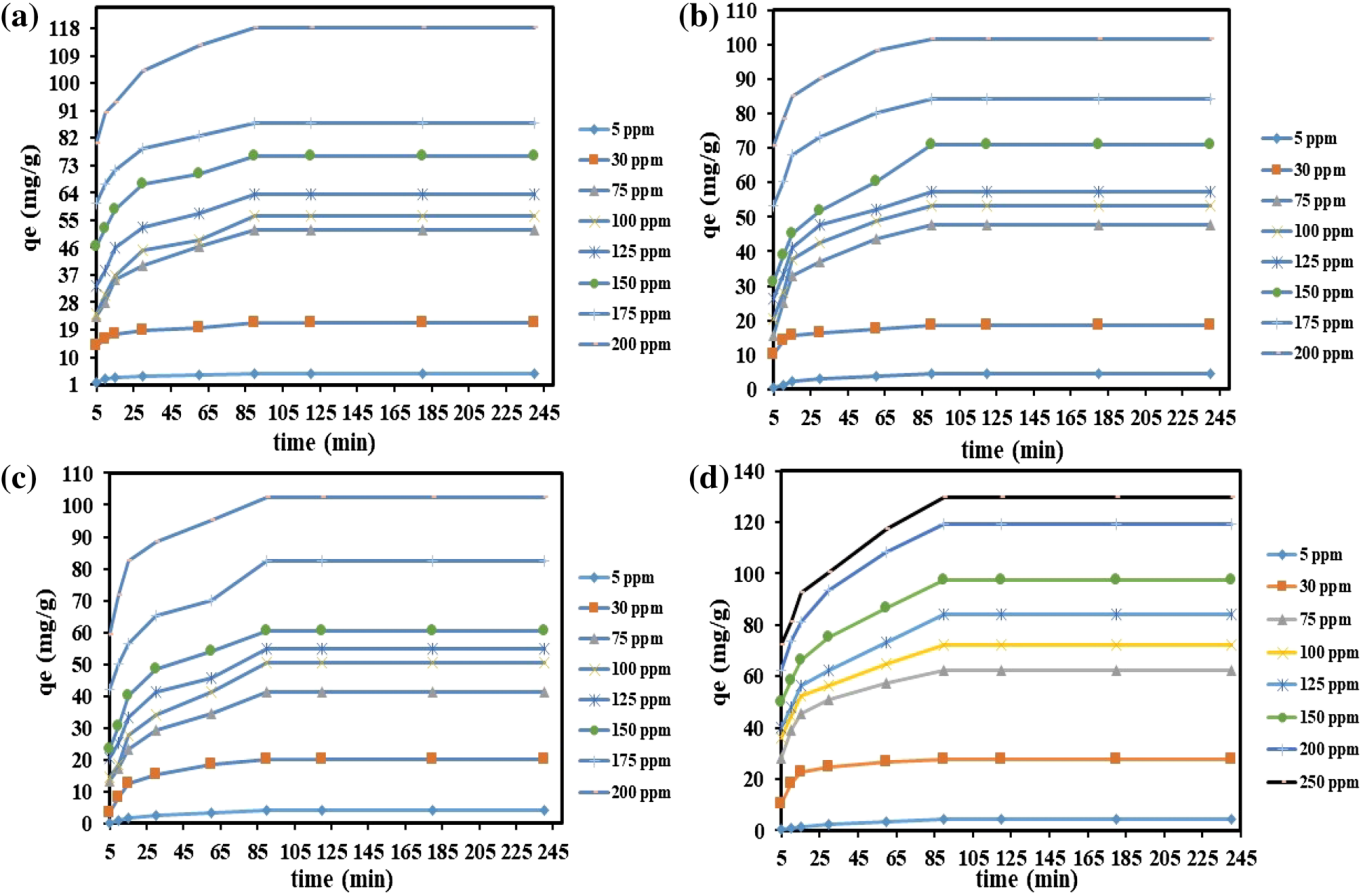
Effect of time and concentration on CR adsorption (**a**), BG adsorption (**b**), MB adsorption (**c**) and MR adsorption (**d**).

Figure 10a–d shows the number of qe values of CR, BG. MB and MR at different ranges of time and concentration. For all the studied dyes, adsorption started quickly due to the empty surface sites in the PA-12/PC nanocomposite, and reached equilibrium at 90 minutes. In 90 minutes, the surface sites of the PA-12/PC nanocomposite captured the dyes, and further no adsorption occurred because the adsorption rate and qe values did not change. There has also been previous research reporting a 90 minute equilibrium time for CR^74^, BG^75^, MB^76^, and MR^77,78^ onto polymeric nanocomposites. It can also be noted that qe values increased with an increase in the concentration of all the studied dyes. With the increase in concentration values, the mole ratio and mass transfer driving force of dye molecules also increase, which led to the uptake high number of dye molecules at the surface of PA-12/PC nanocomposite, and thus qe values also increased.

#### Isotherm modeling

**Figure 11:**
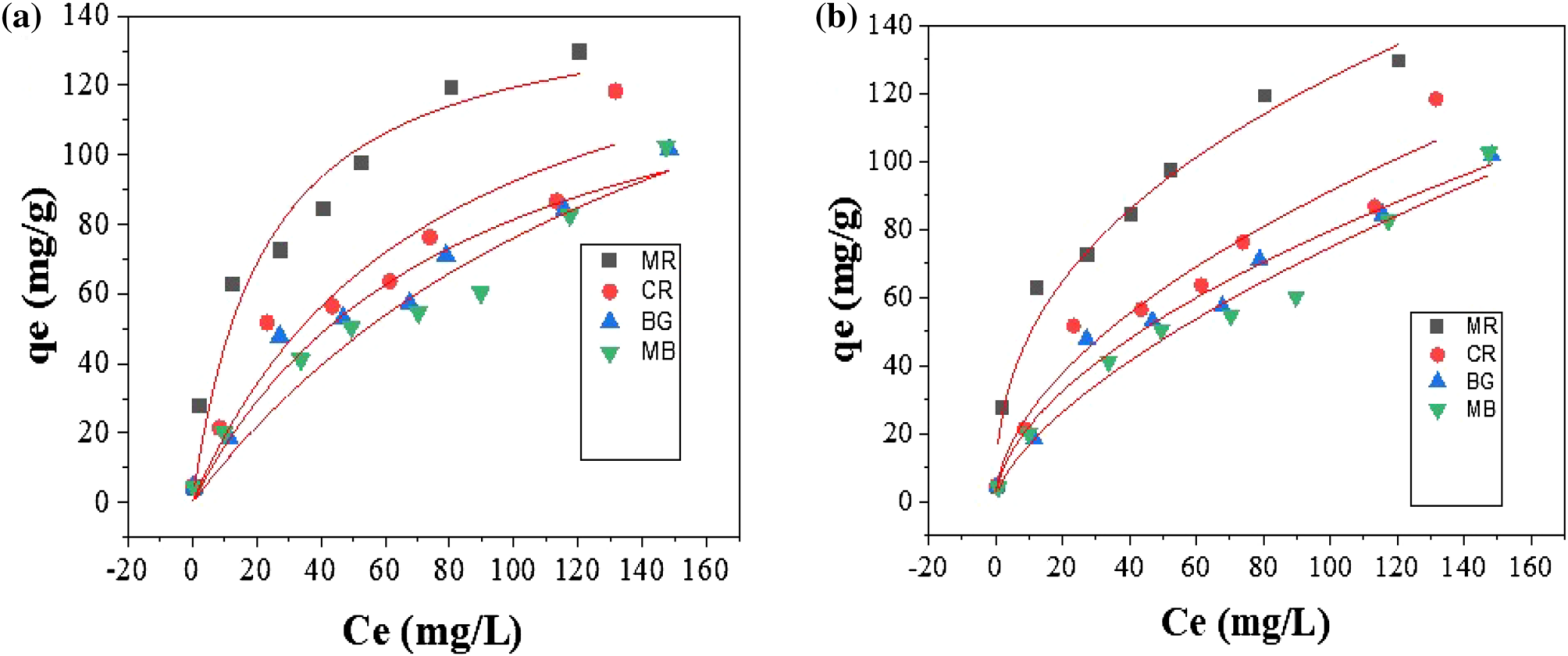
Adsorption isotherm models: Langmuir (**a**), Freundlich (**b**).

The experimental data of dyes adsorption onto PA-12/PC nanocomposite were fitted into non-linear plots of the various isotherm models (Fig. 11) and the respective parameters were calculated by using the generated isotherm equations (Table S1). As shown in Table 1, the Freundlich model was found to be the most applicable method for describing the adsorption of dyes onto the PA-12/PC nanocomposite. This outcome indicates that the functional groups present in the CR, BG, MB, and MR molecules were adsorbed in the form of a multilayer on the heterogeneous surface of PA-12/PC nanocomposite. As also reported in previous studies, the Freundlich model was best fitted for the adsorption of CR^79^, BG^80^, MB^81^, and MR^82^. The values of n are greater than one, indicating easier and more favorable dyes’ adsorption. The positive values of K_f_ and b indicate favorable interactions between CR, BG, MB, MR, and PA-12/PC nanocomposite. Dubinin–Radushkevich (D–R) isotherm parameters were calculated to confirm the nature of the adsorption process. If the value of E comes out to be < 8 kJ/mol, then the adsorption process is considered in physical mode and if it’s equal to or > 8 kJ/mol, then the adsorption process is considered in chemical mode. The calculated E values for all the studied dyes were found to be < 8 kJ/mol which supports the earlier proposed mechanism of multilayered physical adsorption of CR, BG, MB, and MR onto PA-12/PC nanocomposite by electrostatic attraction (Fig. 9). The obtained qm values for the PA-12/PC nanocomposite are in comparison higher than previously reported qm values of various natural and synthetic materials (Table 2)^77–90^ which proves the practical and viable application of the PA-12/PC nanocomposite for the removal of CR, BG, MB and MR from industrial wastewater.

**Table 1:** Adsorption isotherm parameters for the adsorption of dyes.

Dye	Isotherm model	Parameters	Values
CR	Langmuir	q_m_	161.635
		b	0.013
		R^2^	0.9243
	Freundlich	n	1.830
		K_f_	7.37
		R^2^	0.9515
	D–R	q_m_	90.456
		E	0.08
		β	71.00
		R^2^	0.7704
BG	Langmuir	q_m_	148.54
		b	0.0121
		R^2^	0.9624
	Freundlich	n	1.80
		K_f_	6.174
		R^2^	0.9761
	D–R	q_m_	82.363
		E	0.078
		β	80.955
		R^2^	0.8294
MB	Langmuir	q_m_	200.40
		b	0.0061
		R^2^	0.9495
	Freundlich	n	1.548
		K_f_	3.818
		R^2^	0.9677
	D–R	q_m_	87.63
		E	0.05
		β	197.93
		R^2^	0.8063
MR	Langmuir	q_m_	146.41
		b	0.0411
		R^2^	0.9568
	Freundlich	n	2.461
		K_f_	19.19
		R^2^	0.9761
	D–R	q_m_	96.30
		E	0.52
		β	1.810
		R^2^	0.7612

**Table 2:** Comparison of the maximum adsorption capacity of PA-12/PC nanocomposite with various materials used for CR, BG, MB, and MR adsorption.

Dye	Adsorbent	q_m_ (mg/g)	References
CR	Biosynthesized zinc oxide nanoparticles	9.615	^83^
	Silicon carbide nanoparticles	78.74	^84^
	Litchi seed powder	20.49	^85^
	MoS_2_ nanopowder	80.64	^8^
	PA-12/PC nanocomposite	161.63	This study
BG	Chemically modified *Lawsonia inermis* seeds	34.96	^86^
	*Peganum harmala*-L-seeds	35.971	^87^
	ZnO-NP-AC	142.9	^88^
	Chemically modified areca nut husk	18.21	^89^
	PA-12/PC nanocomposite	148.54	This study
MB	*Punica granatum* L. seeds	99.00	^72^
	Purolite (SST 60) cation exchanger	131	^90^
	Luffa actangula carbon	24.84	^91^
	Modified activated carbon	195.8	^92^
	PA-12/PC nanocomposite	200.40	This study
MR	Lemongrass	76.92	^93^
	Eggshell waste	1.66	^94^
	Chitosan/polyacrylamide hydrogel grafted poly(*N*-methylaniline)	6.138	^95^
	Jebel Louka clay	132.30	^96^
	PA-12/PC nanocomposite	146.41	This study

#### Kinetics

To design the dyes adsorption system onto PA-12/PC nanocomposite, the kinetic data of pseudo-first-order (PFO), pseudo-second-order (PSO), Elovich equation, and intraparticle diffusion (IPD) models using equations obtained from linear plots (Table S2) and calculated values of each kinetic parameter (Table 3). It was found that PFO failed to fit the data of dyes’ adsorption and PSO was found to be superior as per higher R^2^ (Fig. S2) and closer (q*ecal*) to (qe*exp*) values. Usually, PSO is found suitable for the data as its equation has less chance to take the effect of the experimental error^97^. The constant values of PFO rate constant (K_1_) and slow decrease in the values of PSO rate constant (K_2_) with an increase in the dye concentrations indicates that equilibrium was achieved at an increasing concentration of dyes'. The intraparticle diffusion plots were used to assess molecular diffusion. The plots were non-linear with a plateau profile and do not pass through the origin; moreover, the unsatisfactory relationship between qt and t1/2 with low R^2^ values suggests that the model was not reasonably fitted (Table 3). The intercept (C) value increased with dye concentration, which indicates an increase in boundary layer thickness. It means that more than one process influenced dyes’ adsorption onto PA-12/PC nanocomposite. Several previous studies have reported that intraparticle diffusion was not the only rate-controlling step in the adsorption process of dye molecules^98,99^. According to Elovich's model linear plots, R^2^ values are mostly  fall < 0.96 for all the dyes’, and therefore the model does not reasonably fit with experimental data in comparison to PSO's model. Table 3 shows that the values of A (adsorption constant) are higher than the values of B (desorption constant) indicating a higher adsorption rate than desorption^100^, which displays the feasibility of the adsorption process. The increasing value of A with increase in concentration supports enriched adsorption of all the studied dyes’ onto PA-12/PC nanocomposite.

**Table 3:** Kinetic parameters for adsorption of dyes on PA-12/PC nanocomposite.

Dye	Kinetic	Parameters	5 mg/L	30 mg/L	75 mg/L	100 mg/L	125 mg/L	150 mg/L	200 mg/L	250 mg/L
CR	PFO	K_1_	0.027	0.026	0.029	0.032	0.023	0.028	0.034	0.032
		qe*cal*	2.800	7.232	29.58	34.60	28.58	29.56	27.36	40.73
		qe*exp*	4.790	21.50	51.73	56.51	63.61	76.13	86.7	118.4
		R^2^	0.908	0.919	0.973	0.982	0.874	0.917	0.971	0.991
	PSO	K_2_	0.022	0.011	0.002	0.001	0.002	0.002	0.003	0.002
		qe*cal*	5.020	21.90	53.76	59.17	65.78	78.12	87.71	120.4
		qe*exp*	4.790	21.50	51.73	56.51	63.76	76.13	86.70	118.4
		R^2^	0.998	0.999	0.999	0.998	0.999	0.999	0.999	0.999
	IPD	K_id_	0.380	0.968	3.745	4.145	3.963	3.918	3.459	5.027
		C	1.355	12.641	17.72	18.17	27.49	40.80	56.07	73.08
		R^2^	0.869	0.924	0.954	0.944	0.944	0.931	0.934	0.964
	Elovich model	A	0.782	11.166	12.331	11.69	21.65	35.19	51.48	66.10
		B	0.810	2.077	7.924	8.970	8.435	8.262	7.149	10.54
		R^2^	0.917	0.944	0.947	0.950	0.946	0.940	0.936	0.950
BG	PFO	K_1_	0.032	0.029	0.029	0.035	0.031	0.022	0.035	0.039
		qe*cal*	4.270	6.273	31.196	32.96	30.26	40.15	32.96	34.85
		qe*exp*	4.550	18.5	47.81	53.1	57.44	71.10	84.33	101.7
		R^2^	0.972	0.841	0.906	0.961	0.935	0.973	0.981	0.989
	PSO	K_2_	0.031	0.0144	0.0023	0.002	0.002	0.001	0.003	0.003
		qe*cal*	4.3	18.86	50	55.24	59.52	74.62	86.20	103.0
		qe*exp*	4.55	18.5	47.81	53.10	57.44	71.10	84.33	101.7
		R^2^	0.994	0.999	0.999	0.999	0.999	0.998	0.999	0.999
	IPD	K_id_	0.537	0.91	4.009	4.195	3.875	3.998	4.075	4.109
		C	− 0.269	10.516	12.412	16.17	21.17	22.11	48.17	65.46
		R^2^	0.941	0.770	0.898	0.900	0.902	0.914	0.934	0.940
	Elovich model	A	− 1.003	9.207	7.005	10.62	16.17	13.92	42.74	60.18
		B	1.122	1.901	8.301	8.648	8.311	11.34	8.435	8.436
		R^2^	0.937	0.856	0.921	0.920	0.934	0.960	0.936	0.935
MB	PFO	K_1_	0.023	0.041	0.025	0.024	0.024	0.032	0.020	0.029
		qe*cal*	4.030	17.49	29.24	37.55	34.28	37.35	38.77	39.69
		qe*exp*	4.170	20.10	41.22	50.5	54.68	60.25	82.6	102.50
		R^2^	0.967	0.977	0.973	0.962	0.930	0.951	0.921	0.923
	PSO	K_2_	0.016	0.003	0.0014	0.001	0.001	0.001	0.001	0.002
		qe*cal*	4.1	21.78	40.05	56.64	57.80	62.89	85.47	105.26
		qe*exp*	4.17	20.1	41.22	50.5	54.68	60.25	82.6	102.5
		R^2^	0.981	0.994	0.998	0.997	0.998	0.999	0.998	0.999
	IPD	K_id_	0.517	2.145	3.726	4.818	4.500	4.879	5.049	5.3131
		C	− 0.710	1.607	6.603	5.442	12.77	16.77	34.02	55.042
		R^2^	0.979	0.882	0.974	0.968	0.957	0.925	0.963	0.8929
	Elovich model	A	− 1.552	− 1.031	0.863	2.170	5.664	10.10	25.25	47.759
		B	1.137	4.335	8.055	10.49	9.784	10.16	11.36	11.159
		R^2^	0.960	0.905	0.956	0.954	0.953	0.934	0.958	0.925
MR	PFO	K_1_	0.032	0.044	0.031	0.026	0.024	0.025	0.029	0.0269
		qe*cal*	4.77	32.43	32.38	36.14	45.47	49.78	62.01	62.37
		qe*exp*	4.23	27.70	62.50	72.5	84.2	97.4	119.2	129.5
		R^2^	0.989	0.899	0.954	0.963	0.978	0.988	0.995	0.9863
	PSO	K_2_	0.003	0.0070	0.002	0.001	0.001	0.001	0.001	0.0011
		qe*cal*	5.37	28.49	64.51	75.18	87.71	101.0	123.4	133.3
		qe*exp*	4.23	27.70	62.5	72.50	84.20	97.40	119.2	129.5
		R^2^	0.951	0.999	0.999	0.999	0.998	0.999	0.999	0.999
	IPD	K_id_	0.55	1.982	4.229	4.618	5.741	6.250	7.629	7.639
		C	− 0.821	11.183	24.80	29.84	30.19	39.03	49.03	58.24
		R^2^	0.994	0.733	0.906	0.956	0.979	0.985	0.985	0.9847
	Elovich model	A	− 1.598	8.820	18.874	22.72	20.97	29.30	37.81	46.49
		B	1.165	3.953	8.848	9.961	12.57	13.56	16.28	16.536
		R^2^	0.950	0.807	0.930	0.958	0.952	0.958	0.958	0.9566

#### Thermodynamics

 With the vant's Hoff plot, the thermodynamic parameters affecting the adsorption of CR, BG, MB, and MR were determined for the PA-12/PC nanocomposite at various temperatures (Table 4 and Fig. S3). Based on the results, the values of − ΔG values increased with temperature, indicating that adsorption of the dyes’ was increased with increasing temperature due to the endothermic process and spontaneous nature. Additionally, the adsorption process was due to the physical adsorption as ΔG values ranged below − 20 kJ/mol^101^. The positive value of ΔH also suggests involvement of the endothermic process and physical adsorption. The positive value of ΔS indicates the increased disorderness at the solid/solution boundary during the favorable adsorption of CR, BG, MB, and MR onto the PA-12/PC nanocomposite.

**Table 4:** Thermodynamic parameters for the adsorption of dyes onto PA-12/PC nanocomposite.

Dye	Temperature (K)	∆G (kJ/mol)	∆H (kJ/molK)	∆S (kJ/mol)	R^2^
CR	288	− 19.32	58.78	0.2678	0.9403
	298	− 22.52			
	308	− 24.30			
BG	288	− 19.25	57.24	0.2624	0.9226
	298	− 22.47			
	308	− 30.01			
MB	288	− 19.186	56.388	0.2593	0.9089
	298	− 22.444			
	308	− 23.993			
MR	288	− 19.184	54.952	0.2545	0.9008
	298	− 22.422			
	308	− 23.891			

#### Desorption and regeneration

**Figure 12:**
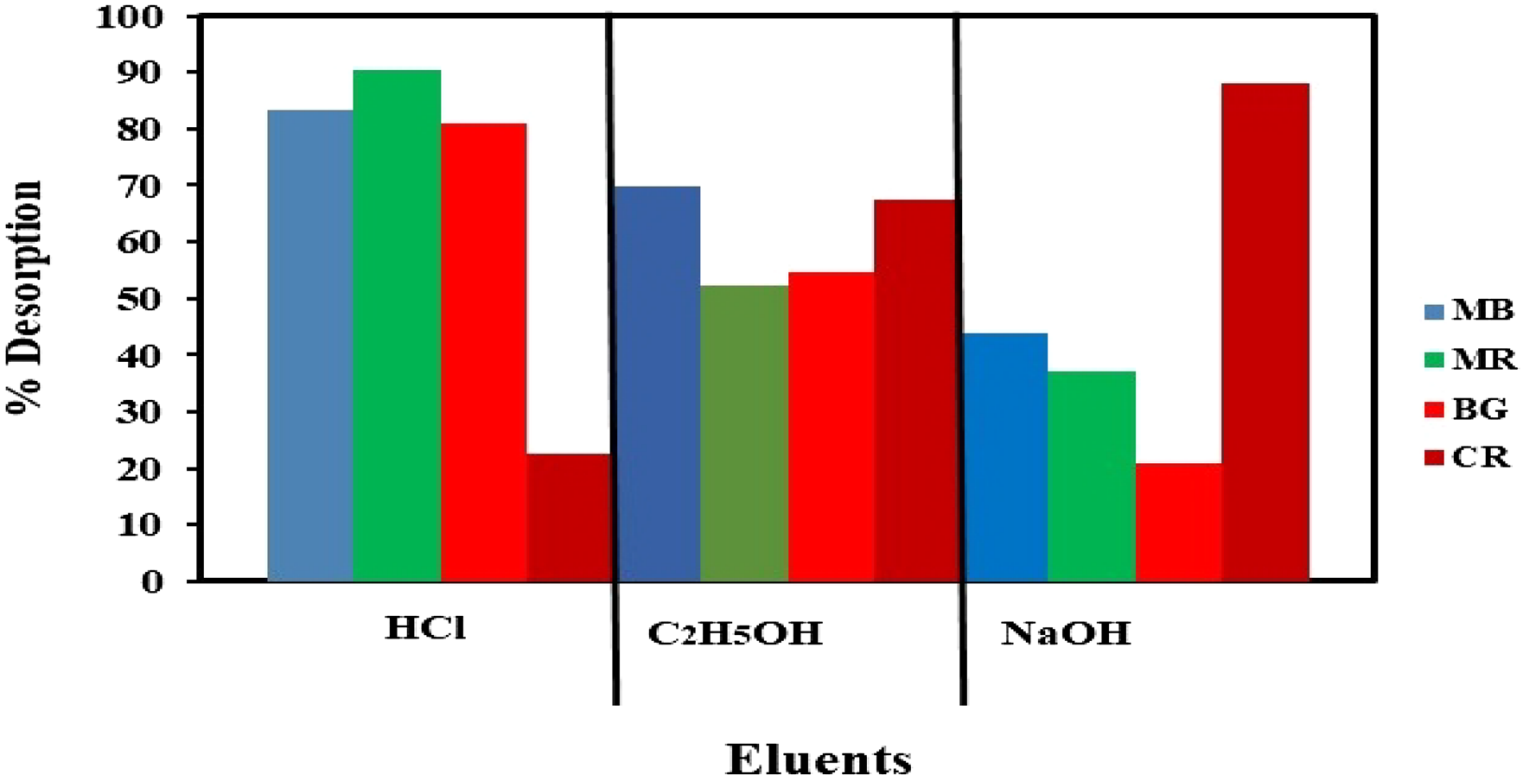
Percentage desorption of dyes using various desorbing eluents.

The chances of dyes adsorbed PA-12/PC nanocomposite retrieval were assessed by desorption experiments using three common desorbing agents (Fig. 12). It was found that a strongly basic solution of sodium hydroxide (NaOH) worked best for CR desorption (88.07%), while hydrochloric acid (HCl) and ethanol (C_2_H_5_OH) solutions desorbed a lesser amount. This outcome supports the result as observed in the pHPZC study that anionic CR was adsorbed onto cationic PA-12/PC nanocomposite. Generally, NaOH has a higher cation exchange capacity and loses its solvation easily during the ion-exchange process. At equilibrium, the quantity of adsorbent is higher than adsorbate^102^, hence when the cations present in the exhausted PA-12/PC nanocomposite were reacted with negatively charged NaOH, the anionic CR desorbed from exhausted PA-12/PC nanocomposite due to electrostatic interaction. This observation confirms the isotherm results that adsorption is dominated by physical adsorption. A strong acid solution of HCl worked best to desorb BG (80.97%), MB (83.43%), and MR (90.37%) due to its solvation efficiency and concentration gradient. Moreover, the acid treatment caused an increase in the protonation on the surface sites and hence made the PA-12/PC nanocomposite less capable to hold adsorbed BG, MB, and MR molecules, causing the release of dyes' during desorption studies. H^+^ promotes the desorption of the cations present in the dye molecules structure i.e., N_2_^+^ in BG, S^+^ in MB, and N^+^ in MR that were previously adsorbed to a negatively charged surface (> pH 5) of PA-12/PC nanocomposite. It can be concluded from desorption results that ion exchange was the main desorption mechanism and physical adsorption was involved in CR, BG, MB, and MR removal by PA-12/PC nanocomposite. Moreover, these results suggest that further research can be conducted to make sure that the four dyes are completely desorbable (> 95%), especially, BG and MB, by increasing the concentration of desorbing agents and changing the parameters such as pH and temperature.

**Figure 13:**
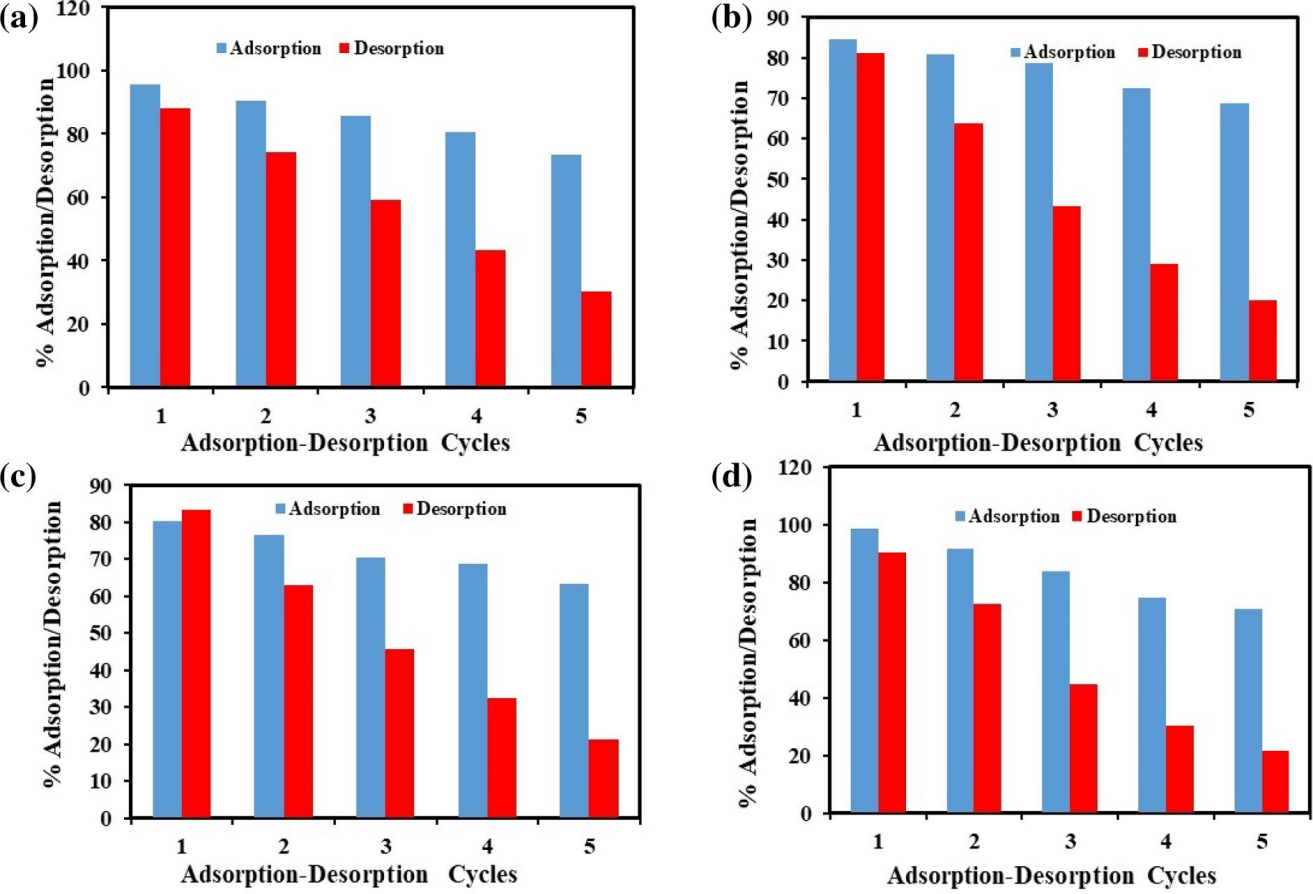
Regeneration performance of PA-12/PC nanocomposite for the adsorption of CV (**a**) BG, (**b**) MB, (**c**) and MR (**d**).

Figure 13a–d shows the percentage desorption efficiency of dyes-loaded PA-12/PC nanocomposite at each regeneration cycle (up to 5). It can be seen that the decrease in percentage removal of CR, BG, MB, and MR is constantly high, which is appropriate for the recycling, practicality, and durability of the PA-12/PC nanocomposite. Also, all the dyes were desorbed easily from the PA-12/PC nanocomposite which attributed to the physical adsorption of CR, BG, MB, and MR removal onto the PA-12/PC nanocomposite. This study revealed that PA-12/PC nanocomposite had 73.6, 68.8, 63.4, and 70.7% stability for CR, BG, MB, and MR adsorption, respectively, after five cycles (Fig. 13a–d). In light of this outcome, it can be concluded that PA-12/PC nanocomposite is economically feasible for the decolorization of industrial wastewater.

## Materials and methods

### Materials

A fresh PA- 12 powder with good mechanical properties, high chemical resistance, and suitable for high-temperature applications was purchased from Sinterit company based in Kraków (Poland). As mentioned PA-12 powder was grey has a granularity of 18–90 μm, a tensile strength of 32 MPa, and a melting point of 185 °C. PC, black color, from Saudi cement company was purchased from a local shop at Al-Majmaah, Saudi Arabia. PC was made of high-quality clinkers and had high compressive strength concrete of more than 40 Mpa. It was produced according to Saudi standards (SASO GSO 1917/2009).

### Preparation of PA-12/PC nanocomposite

PA-12/PC nanocomposite with the loading of PA-12 (5–20 weight percent) was prepared by exfoliated adsorption method^103^. This method is of fundamental importance for the production of clay/polymer nanocomposites with superior material properties^104^. The amount of PA-12 powder and PC was mixed with water and then the colloidal suspension of the PA-12/PC mixture was intensively shaken using a high-speed mixer rotating at 3000 rpm and 80 °C for 30 min. The mixing proportion (weight percent) of PA-12/PC was 95(PA-12):5(PC), 90(PA-12):10(PC), 85(PA-12):15(PC), and 80(PA-12):20(PC) as used in the previous study^49^. As prepared, PA-12/PC nanocomposites of different ratios (weight percents) were then filtered, washed with distilled water, and dried in an oven at 80–90 °C overnight.

### Characterization

FTIR spectroscopy was used to analyze the structure and functionalization of PA-12/PC nanocomposite were analyzed by FTIR spectroscopy in 400–4000 cm^−1^ range by using Perkin Elmer Spectrum IR Version 10.6.1. SEM and EDS along with elemental mapping were used to get the elemental composition and their distribution within PA-12/PC nanocomposite by using JSM-6700F, JEOL, Japan (for SEM), and TESCAN MIRA3, Czech Republic (for EDS). BET technique was used to measure the specific surface area of PA-12/PC nanocomposite by using Quanta Chrome Touch Win™ v1.22. The successful formation of PA-12/PC nanocomposite was confirmed by the XRD pattern obtained with the ALTIMA-IV, RIGAKU X-ray diffractometer. To better understand the deep morphology of PA-12/PC nanocomposite, HRTEM images were obtained.

### Adsorption experiments

As prepared, various ratios of PA-12/PC nanocomposite were initially applied to investigate the removal of dyes from synthetic colored solutions. It was found that among all the prepared PA-12/PC nanocomposites, the most efficient adsorbent for removing CR, BG, MB, and MR was 80(PA-12):20(PC) weight percent ratio. It may be due to the improvement in the surface characteristics and adsorption properties of the prepared nanocomposite after increasing the PA-12 amount. Because of this outcome, the material ratio of 80:20 weight percent was selected as optimal for obtaining the decolorization process by removing dyes (CR, BG, MB, and MR) using the adsorption method. Adsorption studies were carried out in the batch mode by using dark color bottles to avoid any passing of sunlight in it. The adsorbent dose of 0.05 gm. was placed in a beaker and treated with 20 mL of targeted dye solution of 50 mg/L concentration. The solution was then centrifuged at 600 rpm for 20 min and then decanted. Subsequently, the residual concentrations of CR, BG, MB, and MR were measured by a UV–Vis spectrophotometer (Perkin Elmer, USA) at a λmax of 497, 625, 668, and 520 nm respectively. The amount of dye adsorption was calculated using the following equations:

1 $$\mathrm{\% adsorption}=\frac{Ci-Cf}{Ci}\times 100,$$

2 $$\mathrm{qe}=\frac{Ci-Cf}{Ci}\times \frac{V}{m},$$

where C*i* and C*f* are initial and final dye concentrations, respectively, qe is the adsorption capacity of PA-12/PC nanocomposite, V is the volume in L and m is mass in gm.

### Desorption and regeneration

During the desorption experiments, 20 mL of each of the common desorbing agents, including NaOH, HCl, and C_2_H_5_OH were used.  Using the same adsorption-desorption method, the used PA-12/PC nanocomposite was washed with distilled water, dried at 80°C for 2 hours, and reused five times for dyes’ adsorption.

## Conclusion

Easy and facile preparation of PA-12/PC nanocomposite was established throughout this study. SEM images show that PA-12 particles are round in shape while PC particles are angular. A H4 hysteresis loop of type-II isotherm with nearly horizontal and parallel lines over a wide p/p° range has been observed by BET analysis. The BET surface area, total pore volume, and average pore diameter were 2.635 m^2^/g, 0.003 cm^3^/g, and 2.665 nm, respectively. The nanomixture of PA-12 and cement not only had a positive impact on the mechanical properties but also became an excellent adsorbent for removing various dyes and provided novel perspectives for the purification and treatment of drinking water. For the removal of CR, the optimal pH was 4, while it was 6 for the removal of BG, MB, and MR. All dyes were removed after 90 minutes of equilibrium time. The calculated maximum adsorption capacity was 161.63, 148.54, 200.40, and 146.41 mg/g for CR, BG, MB, and MR, respectively. On an industrial scale, the regeneration capability of PA-12/PC nanocomposite for the removal of CR, BG, MB, and MR after five adsorption–desorption cycles has established its usefulness in industrial applications.

## Supplementary Information

Supplementary Tables.

Supplementary Figures.

## Data Availability

The datasets used and/or analyzed during the current study are available from the corresponding author on reasonable request.
