# Supplementary material for: Fabrication of polyamide-12/cement nanocomposite and its testing for different dyes removal from aqueous solution: characterization, adsorption, and regeneration studies
Source: Sci Rep. 2022 Jul 30;12:13144. doi: 10.1038/s41598-022-16977-8 (PMC9338974; doi:10.1038/s41598-022-16977-8)
Supplement: Supplementary file 1 — Supplementary Tables. [file 41598_2022_16977_MOESM1_ESM.docx]

| **Adsorption isotherm** | **Non-linear equation** |
| --- | --- |
|  |  |
| Langmuir | $qe=\frac{q_{m}K_{L}C_{e}}{1+K_{L}C_{e}}$ |
|  | Ce is the equilibrium concentration of the aqueous solution (mg/L), qe is the equilibrium capacity (mg/g), qm is maximum uptake (mg/g), and K_L_ is the Langmuir constant (L/ mg) |
|  |  |
|  |  |
| Freundlich  Dubinin- Radushkevich | $q_{e}=K_{F}C_{e}^{\frac{1}{n}}$  K_F_ and 1/n are the Freundlich constants which suggest the intensity of adsorption and capacity of adsorption, respectively $q_{e}=q_{m}\exp(-\beta\varepsilon^{2})$   *β* is the adsorbate mean free energy per mole,, ∈ is the Polanyi potential. |
|  |  |
|  |  |
|  |  |
|  |  |
|  |  |

**Table** **S1.** Non-linear equations of different adsorption isotherms

**Table** **S2** Linear equations of different kinetic models

| **Adsorption kinetic** | **Linear equation** |
| --- | --- |
|  |  |
| PFO | $\log(q_{e}-q_{t})=\log q_{e}-\frac{k_{1}t}{2.303}$ |
|  | *q_e_* and *q_t_* (mg/g) are the adsorptive removal capacity at equilibrium and variable time (*t*), k_1_ is the rate constant for PFO |
|  |  |
|  |  |
| PSO  Intraparticle Diffusion  Elovich Model | $\frac{t}{q_{t}}=\frac{1}{k_{2}q_{e}^{2}}+\frac{t}{q_{e}}$ k_2_  is the rate constant for PFO $q_{t}=K_{id}t^{1/2}+C$   K_id_ is the intraparticle diffusion constant and C is the intercept. $q_{t}=A+B\ln T$ A and B are Elovich constants |
|  |  |
|  |  |
|  |  |
|  |  |
|  |  |
|  |  |
