# Supplementary material for: Fabrication of polyamide-12/cement nanocomposite and its testing for different dyes removal from aqueous solution: characterization, adsorption, and regeneration studies
Source: Sci Rep. 2022 Jul 30;12:13144. doi: 10.1038/s41598-022-16977-8 (PMC9338974; doi:10.1038/s41598-022-16977-8)
Supplement: Supplementary file 2 — Supplementary Figures. [file 41598_2022_16977_MOESM2_ESM.docx]

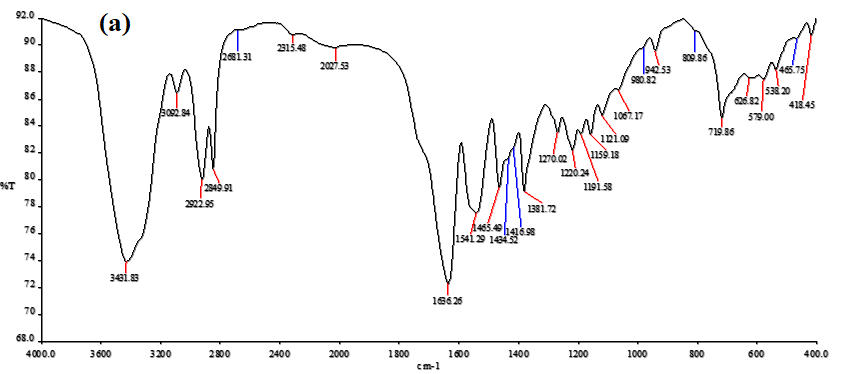


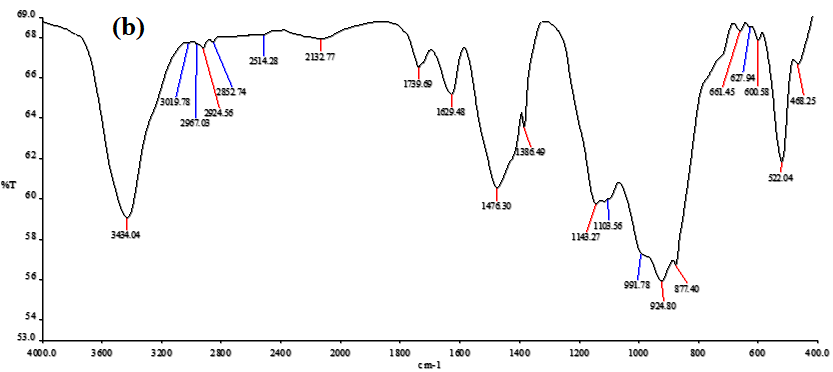


**Fig. S1** FT-IR spectra of PA-12 (a) and PC (b)


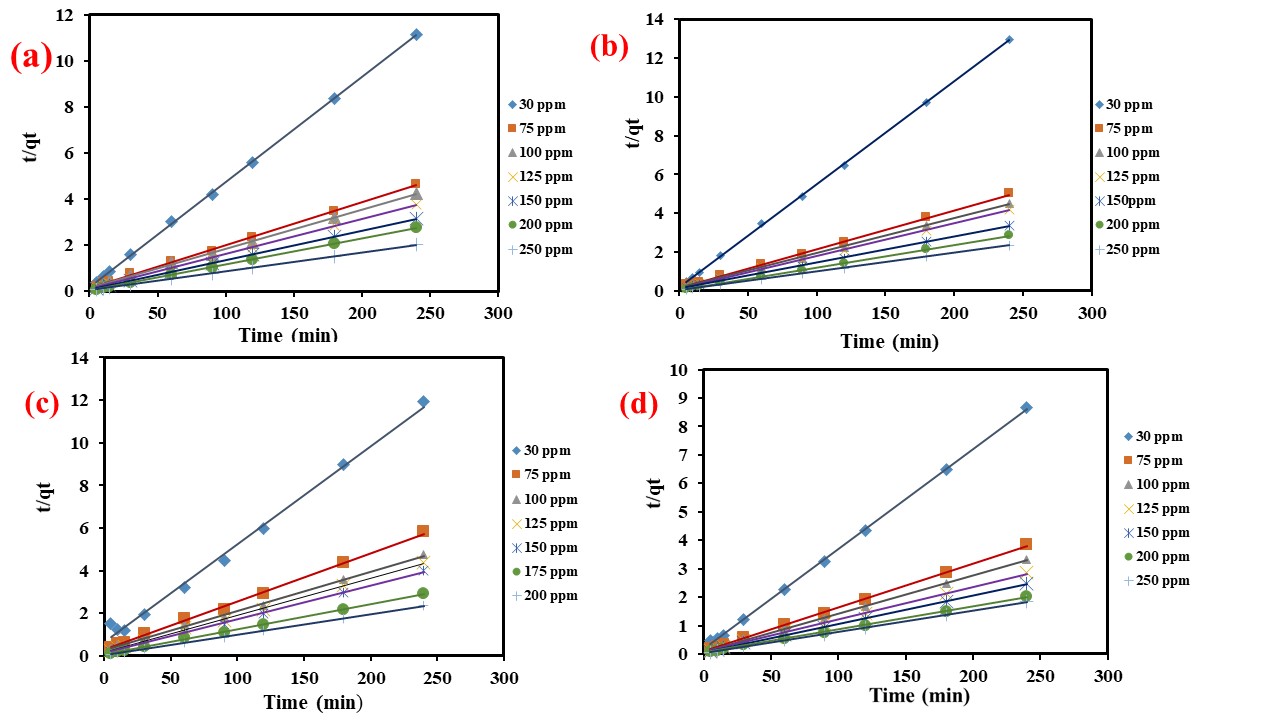


**Fig. S2** Pseudo-second-order-kinetic model: CR(a) BG (b) MB (c) MR (d)

**Fig. S3** vant’s Hoff Plot
